# Conducting Polymers for the Design of Tactile Sensors

**DOI:** 10.3390/polym14152984

**Published:** 2022-07-23

**Authors:** Urte Samukaite Bubniene, Vilma Ratautaite, Arunas Ramanavicius, Vytautas Bucinskas

**Affiliations:** 1 Department of Mechatronics, Robotics and Digital Manufacturing, Faculty of Mechanics, Vilnius Gediminas Technical University, J. Basanaviciaus Str. 28, LT-03224 Vilnius, Lithuania; vytautas.bucinskas@vilniustech.lt; 2 Department of Nanotechnology, State Research Institute Center for Physical Sciences and Technology, Sauletekio Av. 3, LT-10257 Vilnius, Lithuania; vilma.ratautaite@ftmc.lt; 3 Department of Physical Chemistry, Faculty of Chemistry and Geosciences, Vilnius University, Naugarduko Str. 24, LT-03225 Vilnius, Lithuania

**Keywords:** conducting polymers, tactile sensors, conductivity, polypyrrole (Ppy), polyaniline (PANI), poly(3,4-ethylenedioxythiophene) (PEDOT), polydimethylsiloxane (PDMS), conductive polymers, impedimetric sensor, piezoresistive sensor

## Abstract

This paper provides an overview of the application of conducting polymers (CPs) used in the design of tactile sensors. While conducting polymers can be used as a base in a variety of forms, such as films, particles, matrices, and fillers, the CPs generally remain the same. This paper, first, discusses the chemical and physical properties of conducting polymers. Next, it discusses how these polymers might be involved in the conversion of mechanical effects (such as pressure, force, tension, mass, displacement, deformation, torque, crack, creep, and others) into a change in electrical resistance through a charge transfer mechanism for tactile sensing. Polypyrrole, polyaniline, poly(3,4-ethylenedioxythiophene), polydimethylsiloxane, and polyacetylene, as well as application examples of conducting polymers in tactile sensors, are overviewed. Attention is paid to the additives used in tactile sensor development, together with conducting polymers. There is a long list of additives and composites, used for different purposes, namely: cotton, polyurethane, PDMS, fabric, Ecoflex, Velostat, MXenes, and different forms of carbon such as graphene, MWCNT, etc. Some design aspects of the tactile sensor are highlighted. The charge transfer and operation principles of tactile sensors are discussed. Finally, some methods which have been applied for the design of sensors based on conductive polymers, are reviewed and discussed.

## 1. Introduction

Tactile sensors (TS) were ‘born out’ of the observation of natural processes. Therefore, the stimulation and response patterns applied in the operation of a tactile sensor mimic the processes of signal transmission/registration through the nervous system of the human body. The principle of operation of tactile sensors is generally based on aspects of the action of touch, force, or pressure. TS are generally based on the matrix-based principle, which allows monitoring of the entire specific surface area, rather than just monitoring the pressure at individual points. Many types of sensors including TS are based on the application of semiconducting materials deposited on top of interdigitated electrodes in which a *p*-type region is diffused into an *n*-type base [1,2]. Piezoresistive materials with variable conductivity are the most important part of tactile sensors, therefore, the nature of the material from which this part is composed is very important because it influences the performance of the TS and its operational parameters. Electrically conducting polymers are well-known for their unique properties, and as being an excellent choice for their application in the formation of a sensitive element of various sensors [3]. It has been demonstrated that conducting polymers can be applied in the formation of the piezoresistive layers in tactile sensors [4,5]. Therefore, the synthesis of electrically conducting polymer (CP)-based hetero-structures suitable for various sensing applications is an important research area of material engineering [6]. The charge transfer rate in conducting polymers can be influenced by various factors; therefore, they can be used in the manufacture of various sensors. Some CPs change conductivity when external pressure is applied [7].

In addition, some materials such as graphene [8], graphene-based derivatives [9], and MXenes [10,11] (two-dimensional transition metal carbides and nitrides) are also very sensitive to variations of environmental factors, including pressure [5]. Meanwhile, the incorporation of other conducting or semiconducting structures within a CP-based matrix can advance their performance based on the charge transfer [12]. Therefore, the design of such CP-based hetero-composites opens a broad window for the design of tactile sensors with tailored sensing properties [13]. The most important properties of CP, which can be implemented in piezoresistive sensors, are based on: (i) the ability to synthesize CPs by electrochemical [14] and chemical [15] methods; (ii) the high sensitivity of CP under ambient conditions (i.e., temperature, pH); (iii) mechanical resistivity [16], (iv) good electro-mechanical properties; (v) good inherent charge transport characteristics, which provide electrical conductivity [17]; (vi) the structure of the polymer can be easily modified; (vii) easily adjustable sensitivity by adjusting synthetic variables, within doping, changing polymerization temperature, and other parameters; (viii) the sensor layers may be formed by various deposition methods, which allows miniaturization and mass manufacture of such sensors [18]; and (ix) percolation threshold adjustment (re-arrangement) to conduct electricity [19]. Conducting polymers and various hetero-composites based on CPs can be designed by many different methods; therefore, their conductivity and many other physical properties of these polymers can be well adapted [11]. Consequently, conductive nanosized polymeric materials have become important elements in the application of high-performance pressure-converters [20]. They have unique properties related to the high surface area, small dimensions, and large interaction possibilities [21].

Commonly, conductive polymer nanomaterials, which are used in piezoresistive sensors, consisting of polypyrrole (Ppy) [5], polyaniline (PANI), poly(3,4-ethylenedioxythiophene) (PEDOT), and polydimethylsiloxane (PDMS) [4]. Therefore, the role of conductive polymers in the formation of tactile sensors is emphasized in this work. In this paper, we survey the main conductive polymers, the conductivity of which can be modified easily by various syntheses and doping, and thus adapted to obtain a tactile effect without complex laboratory equipment. These are polymers suitable for use in challenging or unusual conditions (e.g., high temperature) and have an excellent miniaturized and nanoscale potential.

The main aim of this review article is to summarize and discuss the charge transfer mechanism of conducting polymer-based composites, which is suitable for the design of tactile sensors.

## 2. Chemical and Physical Properties of Conducting Polymers

Conducting polymers very often act as efficient hole (*p*-type) conductors because of their conjugated molecular backbone. Moreover, if they are used in solution, then they are surrounded by ions and can act as polyelectrolytes that facilitate ion-based conductivity, therefore, the ability of conjugated polymers to conduct both electronic charge and ions (upon doping) is widely exploited. Although the pristine form of conductive polymers has many limitations, hybridization with other materials and various modifications and variations overcome them. Conjugated polymers are ideal for variations in conductivity: although the electronic charge is distributed along the molecular backbone, ion transfer can be also passed through the bulk [22].

Conductive polymers contain delocalized π-electrons in the backbone of the polymeric chains. Therefore, they have high electrical conductivity and low ionization potential [23]. The conductive polymers are found to be effective applications in sensors, rechargeable batteries, screens, displays, transistors, solar cells, photovoltaic devices, light-emitting diodes, and other areas where high electron affinity is reached. The most important issue in the design of charge-transfer-based devices is the selection of a conductive material or composite with suitable properties. Conductive polymers have unique electrical and electrochemical properties, therefore, changes in the physical and chemical properties (e.g., resistance, electrical capacity, piezoresistive properties) of conductive material of a conductive composite substrate can be monitored using a particular signal transducer system, and electrical conductivity can be examined.

**Figure 1:**
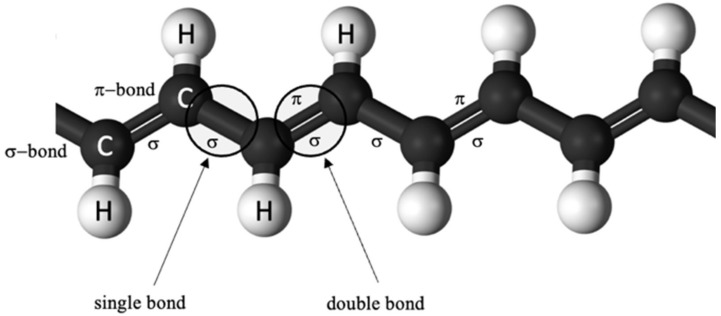
The backbone of polyacetylene (PA) with single and double bonds.

The energy band theory is commonly applied to determine the electrical conductivity by analysis of the material’s electronic structure; however, it does not efficiently explain the CP’s conductive mechanism. In the case of conducting polymers, the conductivity arises from their backbone, i.e., the structure. Conjugated single and double bonds along the molecular backbone determine the CP’s electronic features. The chained backbone part of the CP, based on conjugated bonds, is responsible for its electrical and optical behavior—electrons in these double bonds move along the backbone and allow electricity to flow. Through the rearrangement of these double bonds, the conductivity is subject to change. Localized σ-bond in single and double bonds forms a strong chemical bond (Figure 1). Polyacetylene’s backbone model, because of its simplicity, is used as an example to explain the pathway of the mechanism of conduction in CPs.

## 3. Conducting Polymers as a Charge Transfer Base for Tactile Sensing

Tactile sensors convert mechanical effects such as pressure, force, tension, mass, displacement, deformation, torque, crack, creep, and others, into a change in electrical resistance through a charge transfer mechanism. Polymers are used due to their excellent elasticity and other mechanical properties. There are two main strategies for designing a tactile polymer-based sensor with sufficient charge transfer. Typically, a conductive material, such as metal, is incorporated into the polymeric composite [4]. The second strategy is to use conductive polymers: (i) as a conductive material, and (ii) as a filler rather than as a conductive polymeric composite. For these purposes, conducting polymers can be served as smooth layers obtained by electrochemical deposition methods directly on the electrodes, electrical conducting surfaces, or nanoparticles. In the case of nanoparticle formation of mini-emulsion nanoprecipitation, the microfluidic approach, or self-assembly methods, are also frequently used [24]. Additionally, conducting polymers can be highly conjugated and show metal-like conductivity. In the design of any type of sensor, the selection of the most appropriate monomer to form a sensitive conductive polymer composite is critical. Significant progress has been made in improving the charge transfer of conjugated polymers by changing the chemistry or architecture of the material. CP can possess reversible chemical and physical properties through doping/de-doping processes.

In tactile sensors, conductive polymers can be used as a base in a variety of forms, such as films, particles, matrices, and fillers. A comparison of π-conjugated conductive polymer chemical structure and electrical conductivity is demonstrated in Table 1.

### 3.1. Polypyrrole (Ppy)

**Figure 2:**
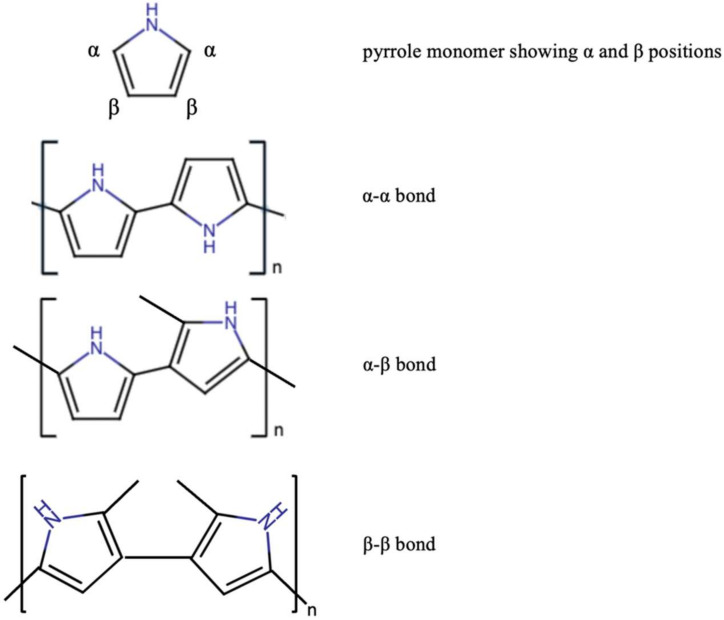
The possible monomer units bonding in Ppy.

Conducting polymer—Ppy—has excellent conductivity in the form of a thin film or bulk material [34]. Ppy is a heterocyclic and positively charged conducting polymer with an oxidized nitrogen based backbone. Ppy is conductive because of the alternating π and σ single and double bonds, which lead to a certain delocalization of electron density in the molecule [35]. Ppy loses its conductivity and a charge when it is overoxidized. The monomer units in Ppy chains are primarily bonded at α-α positions and a minimum amount of pyrrole (Py) monomer is bonded at 2–3 and 3–3 positions (Figure 2) [36,37]. Beta (3) substitution of the pyrrole ring blocks substitution that naturally leads to branching and crosslinking during electro-polymerization. Increased beta substitution, therefore, reduces the opportunities for branching and crosslinking during polymerization [38].

Chemical or electrochemical doping can be used to enhance Ppy conductivity. During doping, Ppy is oxidized, and a π-electron is removed from the neutral Ppy chain, changing its structure. Subsequently, a polaron forms, and with further oxidation, a second electron is eliminated from the Ppy chain, which leads to the formation of a doubly charged bipolaron. After doping, Ppy is converted into the ionic complex consisting of cations and incorporated counterions [34].

The most efficient Ppy polymerization takes place by coupling the electrochemically synthesized cations of the pyrrole monomer radicals through the carbon atoms of the alpha position, followed by the removal of the alpha proton to form a polymer in which all protons in the alpha position are removed.

Electrochemically polymerized Ppy films are typically highly amorphous because of the disordered Ppy chains [39]. Pyrrole units remain intact in the polymer and are linked between the alpha carbon sites.

Conjugated bonds do not render polymeric materials to express high conductivity, but to overcome this issue, doping can be implemented.

Researchers in [5] described a flexible pressure sensor based on Ppy–cotton composites, in which Ppy was grown in situ on cellulose fibers of cotton pads. The optimal Ppy–cotton Pad (PCPs) sensor showed a low detection limit, at about 50 Pa. The in situ vapor growth method ensured the uniform and firm polymerization and coating of pyrrole to the cellulose fibers, in such a way that the PCPs had stable electrical properties. FeCl_3_ was used as an oxidant that promoted the polymerization of pyrrole. FeCl_3_ dissolves in water and forms a homogeneous solution [5]. The authors of this review paper also have developed several methods for the chemical formation of conducting polymers based on oxidation by hydrogen peroxide [40], by Fe(CN)_6_]^4−^/[Fe(CN)_6_]^3−^ based redox cycling [41], and by redox enzyme glucose oxidase [42].

A multifunctional platform based on polyvinylidene fluoride (PVDF)–Ppy reinforced gelatin organohydrogel for a highly recoverable tactile sensor and stretchable strain sensor, was reported [43]. The organohydrogel was synthesized using cation–anion interaction chemistry. The tactile sensor exhibited a sensitivity of 32.39 kPa^−1^ in the wide linear range of 0.1–55 kPa. The strain sensor displayed a gauge factor of 27.8 in the dynamic sensing range of 8.6% to 61%. The sensing mechanism was explained by the piezoelectric polarizability of the β-phase PVDF and the high conductivity of Ppy and gelatin in the flexible organohydrogel [43].

Another study reported a tactile polymer-based sensor, a film of Ppy/dodecylbenzenesulfonic acid (Ppy/DBSA), working in wet conditions [44]. Ppy films were prepared at room temperature (20 ± 2 °C) in dark conditions in a one-compartment electrochemical cell from an aqueous solution of 0.2 M DBSA and 0.2 M pyrrole. Ppy was electrogenerated by applying a constant anodic current. The mechanical sensing characteristics of reactive material films announced the possibility of developing a tactile sensor. The mechanism of operation worked in the following way: if an obstacle was in the muscle’s pathway before reaching the obstacle, the muscle moved freely, but when the muscle touched the obstacle, the mechanical resistance influenced the anodic and cathodic electrochemical reactions occurring in the polymeric Ppy/DBSA film. The working potential of Ppy/DBSA films, oxidized or reduced under constant currents, changed as a function of the mechanical stress [44]. A further study revealed the sensing abilities of the conducting polymers as reactive materials when used to construct tactile artificial muscles. A highly sensitive wearable pressure sensor based on hierarchically patterned Ppy films was reported [45]. Ppy films were composed of three-scale nested surface wrinkling microstructures. These microstructures, combined with stimulus-responsive characteristics and the self-adaptive ability of wrinkling morphologies in Ppy films, offered the as-fabricated piezoresistive pressure sensors a high sensitivity (19.32 kPa^−1^), a low detection limit (1 Pa), an ultrafast response (20 ms), and excellent durability and stability of more than 1000 circles [45]. Another study [46] characterized a wearable pressure sensor based on Ppy as a coating material for an elastic surface—polyurethane. The conductive foams were prepared by in situ chemical polymerization of pyrrole, to retain the elasticity present in the foam substrate. A piece of polyurethane was soaked in the polymerization solution (pyrrole, naphthalene di-sulphonic acid (NDSA), and ferric chloride). The conductance of the conducting foam was found to change linearly with the force applied. The electrical conductance of 1.25 mS/cm was achieved. The responses of the conducting foam to the various weights placed onto the foam (or force applied) were investigated. The weight compressed the conducting foam, to reduce the overall length of the material and increase conductivity.

### 3.2. Poly(3,4-ethylenedioxythiophene) (PEDOT)

PEDOT is a conjugated polymer with many attractive properties required for tactile sensors—good conductivity, excellent flexibility, and good chemical stability [47]. 3,4-ethylenedioxythiophene (EDOT) monomer is used to synthesize PEDOT for in situ polymerization. The most often used methods for polymerization are electropolymerization (EP), oxidative chemical vapor deposition (OCVD), and vapor phase polymerization (VPP).

A study [48] introduced an in situ fabrication method of highly conductive PEDOT films. The process involved the sequential deposition of oxidant V_2_O_5_ and monomers. V_2_O_5_ may change the morphology of PEDOT chains from granular to nanofibrillar, for better electrical connectivity. The obtained PEDOT films possessed good crystallinity and a high doping level, with carrier concentration three orders of magnitude higher than that of the commercial product of PEDOT:PSS. The electrical conductivity of the as-cast PEDOT film reached up to 1420 S/cm. In addition, the synthesis method was fully compatible with large-scale printing techniques. These PEDOT-based conducting films enabled the creation of flexible touch sensors, which demonstrated superior flexibility and sensitivity [48].

MXenes and MX-phase are both very sensitive to various environmental factors [10], including pressure [11]. In [49], a 3D porous Ti_3_C_2_T_x_ (MXene)/PEDOT polystyrene sulfonate (PEDOT:PSS) composite aerogel (MPCA) with a controllable patterning property utilizing the Cu-assisted electrogelation method, was fabricated. The prepared composite aerogel could be assembled into pressure sensors for wearable physical monitoring, and high-resolution sensor microarrays for robotic tactile sensing. The multi-interactions between MXene and PEDOT: PSS enabled the MPCA to have a stable 3D conductive network, which consequently enhanced both the mechanical flexibility and the piezoresistive property of the MPCA. The fabricated pressure sensor demonstrated high sensitivity (26.65 kPa^−1^ within 0–2 kPa), fast response capability (106 ms), and excellent stability [49].

In [50], a type of haptic device was described, that delivered two modes of stimulation—mechanical and electrical. A highly plasticized conductive polymer, based on PEDOT blended with elastomeric polyurethane (PU) was used to achieve an “electropneumotactile” device. The mechanical response of the composite electrodes was highly dependent on the loading fraction of PU. Electrical sensitivity to strain decreased as the loading fraction of PU increased, for example, films with loading fractions of 2.5, 5.0, and 10.0 wt% PU exhibited conductivities of 27, 1.6, and 0.075 S cm^−1^, respectively, (film thicknesses were 360 nm, 415 nm, and 1.02 μm, respectively). Mechanical and electrical stimulation was achieved by combining a stretchable conductor with a pneumatic actuator [50].

In [51], a piezoconductive sensor by a semiconducting polymer, PEDOT, doped with tosylate ions (Tos) thin films, was reported. The examined piezoconductive response of the PEDOT:Tos thin films suggested that the response originated from the deformation of the PEDOT crystal cells and the stretched π–π distances induced by Tos. A negative piezoconductive effect was observed under the pressure [51]. This phenomenon is explained by the piezoconductive response of the value of *R* estimation—as the doping level of Tos was larger than 27% (in this particular research), the *R* was smaller than zero, which indicated that the PEDOT:Tos thin films possessed a negative piezoconductive effect.

The tactile sensing mode can be applied for power generation elements [52]. PEDOT conductive polymer and polyvinylidene difluoride (PVDF) piezoelectric polymer thin films were formed by the dip-coating method. PEDOT solution was prepared by adding ethylene glycol to improve electric conductivity, and dodecylbenzenesulfonic acid to improve the density of the film. Thus, the coating surface was treated with O_2_ plasma to improve the wettability of the PEDOT aqueous solution. The piezoelectric principle is suitable for converting instantaneous force into electricity—PEDOT and PVDF films formed by dip-coating work as electrodes and piezoelectric, respectively [52].

### 3.3. Polyaniline (PANI)

Many researchers working in the field of tactile sensors have focused on simulating tactile perception closer to natural skin, but the challenge remains in developing sensors with high sensitivity over a wide pressure range operating in robotics, mechanical systems, and the wearable tech industry of human–machine interfaces. PANI is a conductive organic polymer of the semi-flexible rod polymer family. Composites of PANI can be easily tuned within a wide domain of shape, size, and structures; therefore, it is widely used in tactile sensors. Doping with acids improves electroactive behavior [53].

In [54], the authors reported a solvent exchange strategy to prepare ultrafine PANI fibers (UFPFs). The obtained UFPFs had a small diameter below 5 µm, mechanical strength of 1080 ± 71 MPa, a high area capacitance above 1008 mF cm^−2^, and a charge storage capacity of 5.25 × 10^4^ mC cm^−2^. An ultrathin all-solid organic electrochemical transistor (OECT) with less than 1 V driving voltage was examined. It essentially amplified drain-source electrical signals with low power consumption and responded to vertical pressure and horizontal friction forces at different levels [54]. The all-solid OECT responded to mechanical deformation as a tactile sensor.

A highly sensitive pressure sensor with a wide linear range was developed in [55]. A hybridized nanofibrous membrane-based pressure sensor was proposed with improved sensitivity and working range. As a primary sensing material, PANI-nanospines were uniformly deposited throughout a hybrid hierarchical nanofiber to achieve high sensitivity (179.1 kPa^−1^) with a low detection limit (1.2 Pa). Additionally, the highly deformable hierarchical nanofibrous membranes enabled a linear response over a broad pressure range (0–50 kPa) with high durability (10,000 cycles).

PANI-based nanofiber wrapped nonwoven fabric was used as the active material to construct pressure sensors in [56]. Due to the unique hierarchical structures, the large surface roughness of the PANI-coated fabric, and the high conductivity of the interdigitated textile electrodes, the obtained pressure sensor showed ultrahigh sensitivity of 46.48 kPa^−1^ in a wide linear range (<4.5 kPa), rapid response/relaxation time (7/16 ms) and low detection limit (0.46 Pa).

Various strategies have been used to construct conductive polymer composites for tactile sensors with low percolation thresholds, good charge transfer patterns, and high mechanical properties. Paper [56] presents a strategy for the preparation of PANI@cellulose nanowiskers (CNs)/natural rubber (NR) nanocomposites with a 3D hierarchical multiscale structure. Specifically, PANI was synthesized in situ on the surface of a CN biotemplate to form PANI@CNs nanohybrids. The electrical conductivity of PANI@CNs/NR nanocomposites containing 5 phr PANI showed 11 orders of magnitude higher than the PANI/NR composites at the same loading fraction. The percolation threshold was drastically decreased from 8.0 to 3.6 vol% [56]. The charge transfer pathway was enhanced by forming a 3D hierarchical multiscale network in a natural rubber matrix. The NR latex was introduced into the PANI@CNs nanohybrids suspension to enable the self-assembly of the PANI@CNs nanohybrids onto the NR latex microspheres. During the co-coagulation process, PANI@CNs nanohybrids were selectively located in the interstitial space between NR microspheres and organized into a 3D hierarchical structure [56], decreasing the percolation threshold.

A stretchable array of highly sensitive pressure sensors was reported in [57]. The proposed pressure sensor consisted of the top layer of Au-deposited PDMS micropillars and the bottom layer of conductive PANI nanofibers on a PET substrate. The tactile effect was achieved by the changes in contact resistance between Au-coated micropillars and PANI, according to varying pressure. This sensor exhibited a sensitivity of 2.0 kPa^−1^ in the pressure range below 0.22 kPa, a low detection limit of 15 Pa, a fast response time of 50 ms, and high stability over 10,000 cycles of pressure loading/unloading with a low operating voltage of 1.0 V [57].

Controllably aligned PANI-based composite films were prepared in [58]. The sensor was fabricated as follows: Fe_3_O_4_ nanoparticles were placed on the surface of silver nanowires (Ag-NWs) (Fe_3_O_4_@Ag-NWs). Controllably-aligned Fe_3_O_4_@Ag nanowire Ag-NW/PANI composite films were then prepared under a magnetic field of 26–42 mT. As a result, the as-prepared Fe_3_O_4_@Ag-NW/PANI composite films showed an excellent electrical conductivity from 5.5 × 10^2^ to 4.1 × 10^3^ S cm^−1^, and a controllable electrical conductivity anisotropy from 1.1 to 6.7. The anisotropic responsive behavior of the Fe_3_O_4_@Ag-NW/PANI composite film makes it an ideal candidate for the fabrication of multidimensional pressure sensors.

Nano-composites based on PANI modified by carbon nanomaterials are often used in sensor design [59,60]. A reduced graphene oxide (rGO)/PANI wrapped sponge was prepared via rGO coating and the in situ synthesis of PANI nanowires (PANI NWs) on the backbones of the sponge for the fabrication of pressure sensors [61]. From the as-prepared flexible sensor, tunable sensitivity (0.042 to 0.152 kPa^−1^), wide working range (0–27 kPa), fast response (~96 ms), high current output (~300 μA at 1 V), frequency-dependent performance reliable repeatability (~9000 cycles), and stable signal waveform output was obtained [61].

Recently, a study demonstrated that PANI film with greater hydrophilicity showed better supercapacitance, as compared with lower hydrophilicity. Supercapacitance makes tactile sensors a mutually beneficial engineered solution. The most hydrophilic nano-nest of PANI electrodes were synthesized by using electrochemical polymerization at room temperature. Experiments showed that the polyaniline material had very good stability and with a maximum capacitance of 757 F g^−1^ and minimum capacitance of 412 F g^−1^ in 1 M H_2_SO_4_ electrolyte within the potential windows of −200 to 800 mV vs. SCE at 5 mV s^−1^ scan rate [62]. PANI is one of the most promising contenders for a high-performance electrochemical supercapacitor due to its excellent high charge–discharge rate, and better columbic efficiency, useful for sensorics applications in the engineering market and in mass production.

The well-defined area is a very important aspect for a tactile sensor. The research of [63] revealed that the PANI nanorice electrode reached 17.33 W h kg^−1^ energy density and 480 W kg^−1^ power density due to significant inner charge contribution (68.28 C g^−1^), compared with outer charge [63]. Such supercapacitors store energy through reversible ion adsorption to facilitate specific high surface area onto active materials, such as a tactile film layer.

### 3.4. Polyacetylene (PA)

The dissemination of the idea that halogen-doped PA (-CH=CH-)_n_ shows high electrical conductivity, led to the 2000 Nobel Prize in Chemistry award. PA was the first polymer exhibiting high conductivity, comparable with metals if exposed to some oxidizing agents. Oxidation forms charge carriers on the conjugated polyene structure [64]. The conductivity of polyacetylene-based films highly depends on the orientation, where films can be oriented by the stretching method. The tensile/stretching method is very important in the production of tactile sensors, as it can significantly increase the response and sensitivity of the sensor with inexpensive and simple technology. To improve the performance of stretchable/tactile sensors, some enhanced structures and morphologies can be designed, and capacitive and resistive properties can be simultaneously measured. Therefore, sensitivity towards mechanical deformations can be significantly improved. Such an improved technique was developed for the tensile drawing of PA films [65]. After doping, tensile drawing and the associated orientation resulted in a major improvement of both the mechanical properties and the electrical conductivity. Maximum values for the modulus and tensile strength were 50 GPa and 0.9 GPa, respectively. The electrical conductivities of highly drawn thin films, doped with iodine, along the orientation direction, reached values at room temperature of up to 3 × 10^4^ S cm^−1^. Electrical conductivity increased linearly with the modulus and tensile strength [65]. Polymer chain orientation as the implicit variable improved charge transfer.

The work of [66] presents the temperature dependencies of the *current–voltage* characteristics of individual helical PA fibers doped with iodine. Using well-dispersed helical PA fibers, the conductivity of the single fiber was measured. The resistance of helical PA fiber decreased from several tens of GΩ down to 870 KΩ after doping. The room temperature conductance of a doped single fiber immediately before cooling was ~8.4 × 10^−7^ S. As the temperature decreased, the initially ohmic *current*–*voltage* curve revealed the nonlinear behavior [66].

The pressure effect on the transport mechanism and the nature of charge carriers of PA were described in [67]. Electrical measurements under hydrostatic pressure (1–4000 bar) showed that electrical conductivity was closely related to the iodine doping rate (0–14%) of PA films. In pristine PA, conductivity increased with pressure, while it decreased in 14% iodine-doped PA. The most important result was an intermediate doping rate (1.5%). Sweeping the range of pressure, conductivity decreased at the beginning and then increased with higher pressure values [67].

### 3.5. Polydimethylsiloxane (PDMS)

PDMS films are the most popular flexible substrates to integrate into tactile-based composites for flexible electronic or e-skin applications due to their excellent elasticity and biocompatibility. PDMS film with microstructures of high density significantly changes its mechanical properties and charge carrier pathways. The applied external pressure causes elastic deformation of the microstructural properties of PDMS, which accumulates and releases energy reversibly to reduce the effect caused by the elasticity of PDMS viscosity [68]. For an unstructured PDMS film, compression increases the release time and lacks the deformable surface, which is a cause of the mechanical and electrical problems in response to the pressure load. The geometry and shape are very important parameters for charge carriers’ patterns, and such flexible substrates, such as PDMS, can minimize the patterns due to excellent visco–elastic behavior.

A polymer-based tactile sensor with multi-directional sensing was presented in [69]. The proposed sensor was diaphragm-like and consisted of a few polymers, such as a PDMS bump, a polyimide (PI) substrate, Cr/Au electrode lines for electrical connection, and NiCr piezoresistors. These types of multiple piezoresistor configurations can detect multi-directional forces. The sensing mechanism was based on a piezoresistive effect, in which the resistance of NiCr changes under mechanical load. The PMDS bump positioned at the center of the sensor transferred an applied force to the PI film, and the piezoresistors were differently deformed depending on the magnitude and direction of the force [69].

A graphene/PDMS array with hierarchical architecture for a pressure sensor application was fabricated in [70]. A highly sensitive piezoresistive pressure sensor with a linear response to applied pressure was demonstrated, by using a hierarchical structure and analyzing its effects on high performance. This type of pressure sensor exhibited high sensitivity (8.5 kPa^−1^) in a wide pressure regime (up to relatively high pressure of ≈12 kPa) with excellent linearity of response, a fast response time of 40 ms, a low limit of detection of 1 Pa, and high durability for over 10,000 cycles. This sensor was able to recognize the very low weight of a fairy starflower (equivalent to a pressure of 1 Pa). In addition, a highly transparent and conductive monolayer enabled graphene electrodes in the sensor to be placed properly onto a target position and to operate with a low voltage of only 1 V [70].

A flexible capacitive tactile sensor was proposed for multi-directional force-sensing [71]. The sensor was based on a dielectric layer of a carbon black/PDMS composite and upper and lower electrodes of carbon nanotubes (CNTs)/PDMS composite layer. By changing the ratio of carbon black, the resolution of the carbon black/PDMS composite layer increased at 4 wt% and then decreased. This was explained according to the percolation theory. The prototype with the carbon black/PDMS composite dielectric layer was fabricated and characterized, and it was concluded that the resolution of the carbon sensor could reach 0.1 N within 50 N in a normal direction, and 0.2 N in 0–10 N in a tangential direction, with good stability [71].

Soft tactile sensors based on CNT–PDMS-gel were studied [72]. The sensor was composed of an elastomer, a rectangular conductive body made from a CNT–PDMS-gel composite, and a circuit board. The conductive body was prepared by mixing different wt% of CNT with PDMS pregel solution. The electrical resistance of the composite changed in response to the strain applied. The hysteresis occurred because of the soft material characteristic exhibited by the CNT–PDMS-gel composite and elastomer. The sensitivity of the sensor was calculated during the increasing load period. The result of the sensitivity was *S* = 0.14 mV/N. Push and release tests were conducted, and the conductive body with 1% CNT was chosen due to its high sensitivity, fast response, and stability in reading output [72].

Sensors with a multilayer structure are widely studied for severe crosstalk effect studies. A design for a resistive tactile sensor array with a coplanar electrode layer and isolated sensing elements, which were made from PDMS doped with the multiwalled carbon nanotubes (MWCNTs) for crosstalk suppression, was presented in [73]. The experimental results demonstrated that a 4 wt% of MWCNTs to PDMS was optimal for the sensing materials. The pressure-sensitive layer consisted of three micro-structured layers—two PDMS/MWCNT-based films and one top PDMS-based film, that were bonded together. Due to this three-layer microstructure design, the proposed tactile sensor array showed sensitivity up to −1.10 kPa^−1^, a response time of 29 ms, and reliability in detecting tiny pressures [73].

Artificial intelligence has also been applied to advance the applicability of tactile sensors. A crude CNT-dispersed PDMS pad, with a bias, was applied to the center, and the resultant piezoresistive current detected at several electrodes located on the pad edge was developed. The hold-out dataset test accuracy for the indented location identification reached 98.89% [74]. PDMS material is very appropriate to use to form a large-area tactile sensor substrate that allows instant detection of an embossed or force-applied location, and pressure therein.

### 3.6. Polyethylene (PE)

The use of inter-operable composites significantly improves the performance and mechanical and electrical properties of tactile materials. For example, the use of a force-sensitive material composite of PE and carbon (Velostat^®^ or Linqstat, Desco Industries Inc., Chino, CA, USA) film as sensitive material for tactile sensors showed many advantages, such as small dimensions (thickness of the film was only 0.2 mm), the required shape was achieved using simple production technology, it was possible to produce arrays of the pressure/force sensors, and flexibility of the polymer-based material, which allowed the creation of a sensor of the desired shape and useable in industrial sizes [75].

The outstanding idea was implemented in [76]. To promote the green chemistry and sustainable development of PE plastics for long-term use in electronic devices, [76] presented a strategy to transform PE plastic bags into highly flexible and transparent triboelectric tactile sensors (TES). Liquid-phase electrolytes (LPE) were introduced between the two thin layers of PE to increase electrical power.

The mechanism of the working action of PE/LPE TES is explained by the principles of operation of coupling behavior between contact electrification, electrostatic induction, and ion conduction in an electric double layer. PE/LPE TES showed an optimized signal response of voltage *V*_oc_~1, 6 V, I_sc_~673 nA, and 450 W/cm^2^. The integration of PE/LPE TES with the signal processing circuit further improved the wireless application of the entire tactile sensing system [76].

The use of PE and its derivatives of the family of polyolefin resins can also be used as a basis for modifications. The authors of [77] reported a tactile sensor with a structure consisting of a pair of compliant conductive plates bonded to graphene films (GF) on the surface layer of a PE terephthalate (PET) substrate and a transparent elastic adhesive sandwiched between the electrodes. The resistance of such a device was directly related to the tactile sensation. It should be noted that the rate of change of resistance was up to 420% when the displacement was changed by 25 mm. The tactile sensor had a high sensitivity of 0.143 mm^−1^, a long lifetime of 14,000 cyclic load tests, and fast response of 0.3 ms. The electrical signals of the tactile sensors were not affected by interference signals such as vertical displacement, stress magnitude, stress range, or bending deformation [77]. A comparison of tactile sensors according to their flexibility, stretchability, and self-healing properties is demonstrated in Table 2.

## 4. Design Aspects of Sensors

**Figure 3:**
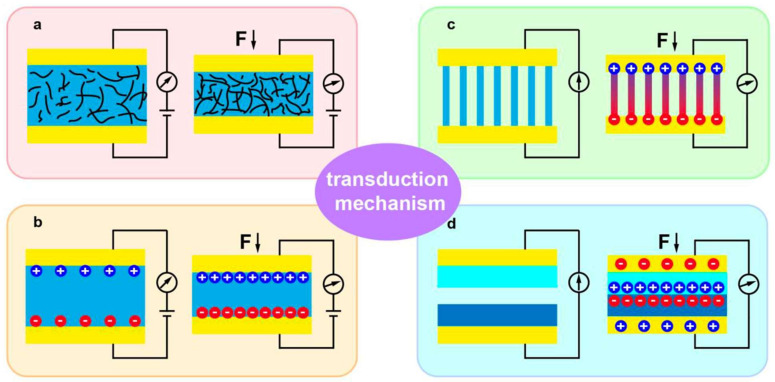
Schematic illustrations of the four typical transduction mechanisms: (**a**) piezoresistive, (**b**) capacitive, (**c**) piezoelectric, and (**d**) triboelectric sensing [79].

The idea of the tactile sensor is—at the same time—very similar and very different from any other type of electrochemical sensor. The focus of this review is dedicated to conducting polymers. Conducting polymers are widely used in the design of electrochemical sensors. The key difference between any other electrochemical sensor and tactile sensors is based on the registered signal. The signal that is registered by any other electrochemical sensor is current, voltage, or resistance. Tactile sensors are classified mainly by the four typical transduction mechanisms. These mechanisms are piezoresistive, capacitive, piezoelectric, and triboelectric sensing (Figure 3) [79]. The key benefits of the piezoresistive tactile sensors are their high sensitivity, simplicity of structure, and low cost. Their applications are mainly distinguished as force, displacement, weight, pressure, flow, torque, and acceleration sensors [80]. The working principle of capacitive tactile sensors is based on the measurements of the dielectric constant, which is related to capacitance by the equation: *C* = *εA/d*, where *C* is the capacitance, *ε* is the dielectric constant, *A* is the area, and *d* is the distance between electrodes. Such tactile sensors are characterized as sensitive, able to be used for static force measurement and having a low power consumption [79]. The final result depends on the normal and shear stress, or contact pressure sensing [81,82]. Regarding the aim of this review, piezoelectric sensors are less interesting, because of restrictions in the creation of high-performance tactile sensors arising due to the low piezoelectric constant of piezoelectric polymers [79]. The working principle of triboelectric tactile sensors is based on the generation of positive and negative electrostatic charges of two different materials in contact with each other via an external force [79]. Among these four types of transduction mechanisms in tactile sensors, the most common is the piezoresistive mechanism.

In comparison with electrochemical sensors, tactile sensors are more compatible with a higher level of machine learning or artificial intelligence for signal interpretation and evaluation.

Some studies concluded that the ionic diffusion coefficient decreases with the thickness of the polymer film, and it is slightly influenced by the electrolyte concentration. Focusing on the cationic doping of Ppy, it was possible to observe a change in the diffusion speed as a function of the ionic radius of the dopant [83]. Such a conclusion was obtained despite different polymerization methods, namely, galvanostatic, potentiostatic, and potentiodynamic, being applied for Ppy layer production. Pulsed current polymerization undoubtedly leads to more compact and adherent films. It was found that the reversibility of the redox process of the films decreased, and the thickness could not be simply related to the polymerization charge [83]. This observation is very important for different applications of conducting polymers, especially for sensors, where a thin and compact layer of polymer is needed.

The results of the electrochemical quartz microbalance (EQCM) experiments confirm that both anions and cations participate in the overall ionic charge transport during the redox electrochemistry of Ppy films. The relative contribution of different substances to the solution–polymer charge exchange varies as a function of potential, i.e., with the degree of oxidation of Ppy, and depends upon the nature and concentration of the electrolyte solution, as well as the film thickness. The phenomena of swelling may be explained by changes in the film morphology resulting from interactions between the polymer and ions, and solvent molecules [84].

There are mainly two processes that govern the current: ‘faradaic’ and ‘capacitive’ [85]. The same study suggested these statements: (i) in the simplest analysis of the form of the CV curve, the faradaic processes are indicated by the peaks, and the plateau indicates the capacitive processes, however, such a simple understanding does not work in all systems and the CV curves represent a superposition of several different processes; and (ii) according to the EIS method, the ‘capacitive’ behavior of the low-frequency impedance, Z ≅ (jωC)^−1^, is sometimes interpreted as an indication of the ‘double-layer’ type of charging. However, this simple interpretation might be confusing due to the possibility of charging the film to different levels as a function of the applied potential, with no direct current passage [85]. However, the chemical nature of all electronic species (such as ‘polarons’, etc.) participating in the formation of the ‘double layers’ at the surface of the macro- or ‘nanopores’ inside the film, is quite identical to that of the “bulk polymer species”. Therefore, all these electronic species must give a similar contribution to the optical signal [86].

The charge transfer at the interface of Ppy and diamond was evaluated by Cermak et al. [87,88,89,90]. They concluded that covalent bonding between Ppy and the diamond electrode was obtained. Additionally, they stated that the charge transport in conjugated polymers was much more probable along the polymer chains than hopping to neighboring chains [88]. A comparison of Ppy—Pt(111) and Ppy—C(111):H interfaces showed that binding energies showed that the Ppy–Pt interfaces were energetically more stable than the Ppy—C:H contacts [91]. In the same study, the values of the charge transfer were calculated. It was found that the charge transfer values were not affected by the number of bonds formed between the polymer and the Pt electrode; meanwhile this value in the case of the Ppy—C:H interface was two times lower.

Typically, the charging/discharging process of conducting polymers is highly related to the doping/de-doping process [92]. In the case of doping processes, conducting polymers are typically charged. Sadly, the value of the charge transfer in the conducting polymer is dependent on the level of polymer degradation. The investigation of Ppy degradation was performed employing electrochemical impedance spectroscopy [93]. For this purpose, the Ppy was overoxidized by potential cycling. After each 10 min of overoxidation, the electrochemical impedance spectra were registered. It was observed that the process of ion insertion in the polymer matrix was damaged, according to these experimental conditions. Due to these processes, the elements of charge transfer in the polymer/solution interface changed (charge transfer resistance *r_ct_* and the charge transfer capacitance *q_ct_*). In the model of another study, the ionic charge transfer across the polymer solution interface could occur far away from an intercalation site in the polymer backbone [94]. In this study, it was assumed that after an ion crossed the solution polymer interface, for some time there remained a charge accumulation that could be described by a capacitive process. In other words, an ion could cross the interface anywhere, but it needed to reach a stable intercalation site after this step.

The synthesis of electrically conductive polymers can be performed chemically or electrochemically. Chemically, monomers are oxidized with oxidizing agents or catalysts. Electrochemical polymerization involves the direct formation of an electrically conductive polymer, which makes it easier and more accurate to control the thickness and morphology, especially roughness, of the formed film. Enzyme-induced formation [42,95,96] and microorganism-assisted synthesis [97] have also been used, although it is not common in tactile sensor research.

An electrochemical process is a way to obtain polymer films of a certain thickness by controlling the number of cycles or the current flowing through the electrode, giving a certain potential. Such an electropolymerization method can be divided into two categories according to processing: (i) with additional processing steps, using dissolvable templates; (ii) without additional processing steps, i.e., directly.

Electrochemical polymerization of fibrillar PEDOT, in the presence of different molecular weights of polyacrylic acid (PAA) with the addition of LiClO_4_ as a co-dopant on the neural electrode probes, was carried out under galvanostatic conditions, with removal of the residuals by washing in water after the electrochemical polymerization was completed [98]. This study revealed that maximizing the effective surface area of the electrode coating by depositing inherently rough materials made it possible to minimize the electrical impedance.

In [99], electrochemical deposition of Ppy layers on paraffin impregnated graphite electrode (PIGE) was performed using cyclic voltammetry (CV). A PIGE electrode was used because of the possibility of achieving good reproducibility of the surface, in contrast with the bad reproducibility of some metallic electrode surfaces. PIGE provided a high-quality Ppy film with a cauliflower-like structure with direct electropolymerization from the alkaline electrolyte. Oxidative electrochemical polymerization of pyrrole from aqueous solution was investigated by multiple scan CV to elucidate the effect of pyrrole monomer concentration, pH, electrolyte solution stirring rate, and potential scanning rate on pyrrole electrochemical polymerization on the surface of PIGE electrode, and the effect of these parameters on Ppy morphology [99].

The deposition of electrically conductive polymers as a basic layer for sensors plays an important role. Different preparation and surface deposition options give the polymer composite layers unique and compatible structures. Due to the ease with which different required elements can be incorporated into composite materials, conductive polymers have been widely used in many fields, especially in the emerging, important part of new and modern sensors [100].

One more parameter emphasized in the latest articles concerned with tactile sensors, was self-healing ability. This parameter is crucial for the lifetime of a sensor, especially of the tactile sensor, which undergoes repeated mechanical impact in the way of scratching, delamination, bending, stretching, etc. [101]. In long-term applications, the ability of self-healing is highly important to the reduction in electronic waste to meet the requirements of environmental protection. Xiaoqian Su et al. [78] described self-healing tactile sensor development based on the composition of such polymers as a conducting polymer (poly(3,4-ethylenedioxythiophene):poly(styrenesulfonate), PEDOT:PSS) and a soft-polymer (poly(2-acrylamide-2-methyl-1-propanesulfonic acid), PAAMPSA). In such a tactile sensor, PAAMPSA was the polymer that was responsible for the self-healing feature and stretchability of the whole composition of polymers. More polymers have been used to achieve the self-healing effect of the tactile sensor, including hydrogenated 4,4′-methylenediphenyl diisocyanate (HMDI) and aliphatic disulfide bis(2-hydroxyethyl) disulfide (HEDS), that was used by Ruiyuan Liu et al. [102]. A self-healing effect was achieved through the dynamic aliphatic disulfide bonding interactions. Next, a self-healing composite for tactile sensing was obtained in the composition of a self-healing elastomer as a triboelectric layer and a transparent conducting polymer, poly(3,4-ethylenedioxythiophene): polystyrene sulfonate (PEDOT:PSS) [102]. Self-healing properties were also obtained in the combination of polytetramethylene ether glycol (PTMEG), hydrogenated 4,4′-methylenediphenyl diisocyanate (HMDI), and aliphatic disulfide bis(2-hydroxyethyl) disulfide (HEDS). To conclude, the self-healing property of the tactile sensor is a promising way to achieve the highest durability of the tactile sensor, to reduce electronic waste. The comparison of pressure sensing parameters of different tactile sensors is demonstrated in Table 3.

## 5. Charge Transfer and Operation Principles of Tactile Sensors

The four types of operation principles currently used in tactile sensor application are piezoresistive, piezocapacitive, piezoelectric, and triboelectric [103]. These working mechanisms describe the tactile sensor operation that detects mechanical external stimuli. The piezoresistive principle exploits the semi-conductor that is affected by strain-induced modulation of the conduction mechanism. When the sensor undergoes deformation, the resistance is affected, therefore, the output current also changes according to Ohm’s law. The piezocapacitive effect is based on the working mechanism of a capacitor. The output capacitance change is observed when the physical structure of the dielectric changes because of the external force applied. The piezoelectric effect is described by the ability of certain materials to generate an electric charge in response to an applied external mechanical pressure. It is the internal generation reaction of an electric field according to a change in dipole moment. The most important factor in this effect is the change in polarization under the action of an external force [103]. The triboelectric effect specifies contact electrification that occurs when a particular material is charged, after being separated from the surface of another material.

### 5.1. Electrical Transport

Charge carriers are electrons or holes that are formed by modifying the polymer backbone—certain blending or doping agents that act as electron receptors or electron donors in the polymer chain. This creates electron holes in the polymer chain or additional electrons that allow the electric current to move in the conjugated chain. The way electrons flow through polymeric nanocomposites depends on the ability of particles to provide a pathway via the electron tunneling mechanism. An effective conductive network in the nanocomposite material must be formed to inhibit the current flow through it. The electron flow in conducting polymer films highly depends on its structure [104]. It is common for PA to be used as an exemplary model to illustrate the principles of conductivity in conductive polymers, due to its simple chemical structure and extremely high electrical conductivity [105]. The charge transfer is responsive to the physical properties of conductive polymers, and the most effective parameters are CP’s conjugation length, degree of crystallinity, and both intra- and inter-chain interactions [105].

### 5.2. Filler Effects

Reasonable coupling of conducting polymers with other materials can result in new composites of eligible properties and parameters. A rich spectrum of conductivity can be achieved by varying the structural ordering and arrangement of a filler matrix, whether it is a 1D, 2D or 3D framework.

The primary electron transfer mechanism of a polymer composite embedded with 1D conductive subjects can generally be described by percolation theory [19]. The transition of electron flow properties occurs by forming interconnected filler networks at a certain threshold filler concentration. The interface between the polymer and conductive filler [106] also plays an important role.

Two-dimensional conductive subjects are described by tunneling theory, where the transition of electron flow properties occurs in a network of heterogeneous conducting filler with an insulating material matrix system. As a new example, two-dimensional transition metal carbides, commonly known as MXenes, have been exploited as excellent material candidates for use as fillers in polymer nanocomposites [2].

Three-dimensional materials as conductive filler matrixes are examined by the tunneling effect, which can take place by each particle being connected to others electrically rather than geometrically, especially when hopping can occur. The migration of the charge carriers in polymer systems occurs via hopping from an excited state to an adjacent state and can proceed with the flow either by interchain (between polymer chains) or intrachain (along a polymer chain) mechanisms depending on the packing/ordering of the macromolecule [107].

Different fillers can be synthesized in various shapes and mixed to improve the charge transfer path. In [71], researchers used several composites of fillers: three types of carbon nanomaterials were used as conductive fillers, including zero-dimensional (0D) carbon black, 1D CNTs, and 2D graphene. The combination of different conductive fillers was beneficial because it was possible to effectively lower the percolation threshold of composite conductive fillers.

## 6. Conclusions

In tactile sensors, conductive polymers can be used as a base in a variety of forms, such as films, particles, matrices, and fillers. Methods applied in the design of sensors based on conducting polymers are highly dependent on the scope and conditions to be met, whether usage for humans such as e-skin, robotic systems, or mechatronics in industrial use.

Conducting polymers and various hetero-composites based on CPs can be designed by many different methods, and conductivity and many other physical properties of these polymers can be well adapted. Therefore, there is broad scope for new applications of conducting polymers in the design of tactile sensors and other sensing devices.

## Figures and Tables

**Table 1:** π-conjugated conductive polymer chemical structure and electrical conductivity.

π-Conjugated Conductive Polymer	Abbreviation	Formula	Electrical Conductivity (S cm^−1^)	Chemical Structure of π-Conjugated Polymers	Ref.
Polypyrrole	Ppy	[C_4_H_2_NH]_n_	From 42 to 6.4 × 10^−10^	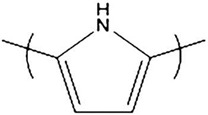	[25]
Poly(3,4-ethylenedioxythiophene)	PEDOT	[C_6_H_4_O_2_S]_n_	6259	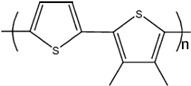	[26]
Polyaniline	PANI	[C_6_H_4_NH]_n_	From 0.1 × 10^−10^ up to 100	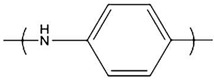	[27]
Polyacetylene	PA	[C_2_H_2_]_n_	>20,000 (obtained after doping with iodine)	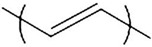	[28]
Polydimethylsiloxane	PDMS	[C_2_H_6_OSi]_n_	From 10^−2^, 0.1 to 7.8 (with the use of fillers)	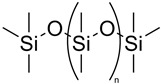	[29,30]
Polyethylene	PE	[C_2_H_4_]_n_	From 0.1 to 100 (with the use of fillers)	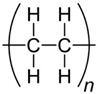	[31,32]

Signal-amplified conjugated polymers for detection on a solid support for a tactile sensor may be acquired as an effective way to boost performance [33].

**Table 2:** A comparison of the tactile sensors according to their flexibility, self-healing or self-recovery properties, and stretchability.

Conducting Polymer	Matrix	Flexibility	Self-Healing or Self-Recovery Properties	Stretchability	Charge Transfer	Ref.
Ppy-cotton	Cotton	+	−	−	Ppy	[5]
PVDF/Ppy/gelatin	Gelatin	+	−	+	Ppy	[43]
Ppy-polyurethane foam	Polyurethane	−	−	−	Ppy	[46]
Ppy-PDMS	PDMS	+	−	−	Ppy	[45]
MXene/(PEDOT: PSS)	PODOT:PSS	+	−	−	MXene, PEDOT	[49]
PEDOT:Tos (tosylate)	−	–	−	−	PEDOT, TOS	[51]
PANI-nanospines of MXene/cellulose on PDMS	PDMS	+	−	−	PANI, MXene	[55]
PANI on nonwoven fabric and cotton substrates	Nonwoven fabric and cotton substrates	+	−	−	PANI	[56]
PANI on PET and Au-coated PDMS, Ecoflex	PDMS and Ecoflex	+	−	+	PANI, Au	[57]
Graphene on PDMS	PDMS	+	−	−	Graphene	[70]
4 wt% of MWCNTs to PDMS	PDMS	+	−	−	MWCNTs	[73]
PEDOT:PSS and PAAMPSA	PAAMPSA	+	+	+	PEDOT:PSS	[78]

**Table 3:** Comparison of pressure sensing parameters of different tactile sensors.

Material	Sensing Mechanism	Sensitivity	Detection Limit	Response and Recovery Times	Ref.
Ppy–cotton	Piezoresistive	4.48 kPa^−1^	50 Pa	220 ms, 240 ms	[5]
PVDF/Ppy/gelatin	Photodetector pressure, and strain sensor	32.39 kPa^−1^	-	0.2 s, -	[43]
Ppy-polyurethane foam	Piezoresistive	0.813 N/cm^2^	-	<5 s, <5 s	[46]
Ppy-PDMS	Piezoresistive	19.32 kPa^−1^	1 Pa	20 ms	[45]
MXene/(PEDOT:PSS)	Piezoresistive	26.65 kPa^−1^	-	106 ms	[49]
PANI-nanospines with MXene/cellulose on PDMS	Piezoresistive	179.1 kPa^−1^	1.2 Pa	-	[55]
PANI on nonwoven fabric and cotton substrates	Piezoresistive	46.48 kPa^−1^	0.46 Pa	7 ms, 16 ms	[56]
PANI on PET and Au-coated PDMS, Ecoflex	Piezoconductive	2.0 kPa^−1^	15 Pa	50 ms	[57]
Graphene on PDMS	Piezoresistive	8.5 kPa^−1^	1 Pa	40 ms	[70]
4 wt% of MWCNTs to PDMS	Piezoresistive	1.10 kPa^−1^	-	29 ms	[73]
PEDOT:PSS and PAAMPSA	Piezoresistive	164.5 kPa^−1^	-	19 ms	[78]
